# Linking D-Dimer and haematological parameters among Indian COVID 19 patients

**DOI:** 10.6026/973206300191179

**Published:** 2023-12-31

**Authors:** Rashmi GS Basavaraj, Ravikumar B Malladad, Ratnakar Ashwini, Manasa Reddy

**Affiliations:** 1 Department of Pathology, Haveri Institute of Medical Sciences, Haveri, Karnataka, India; 2 Department of Emergency Medicine, Subbaiah Institute of Medical Sciences, Karnataka, India; 3 Department of General Pathology, KAHER's JNMC, Belagavi, Karnataka, India; 4 Consultant Pathologist, Sri Padma Gastro Care Clinic, Karnataka, India

**Keywords:** COVID-19, D-Dimer, CT and anaemia

## Abstract

The severe acute respiratory syndrome coronavirus-2 (SARS-CoV-2) outbreak continues to place a significant strain on healthcare systems, economies, and patient management. Therefore, it is of interest to evaluate the role of D-Dimer and haematological parameters to identify severity and outcome of COVID 19 patients. Total 100 cases diagnosed with COVID 19 were recruited in the study and followed up for 6 months. The subjects were grouped into 2, Group 1: Newly Diagnosed COVID 19 Patients and Group 2: After 6 months of follow up COVID 19 Patients. We analyzed Hb, RBCs, WBCs, PT, APTT and D-Dimer and also, we taken CT values of the study subjects. A statistical analysis was done by using SPSS version 20.0. The WBCs and haemoglobin mean values are shown significant values between the study subjects, respectively with p-values < 0.001**. The PT and APTT significantly increased in newly diagnosed COVID 19 patients when compared to after 6 months of follow up at p-value < 0.001**. There was a positive correlation of WBCs, PT, APTT (r= 0.458, 526, 509) with D-Dimer and negatively correlated RBCS, Hb, CT (-0.056, 321, 526, 353), respectively at p < 0.001**. Thus, low platelet, high d-dimer, and fibrinogen may serve as risk markers for the progression of COVID-19 severity. Hence, COVID-19 patients may experience anaemia-related consequences as hypoxia, coronary and pulmonary failure due to low Hb concentration. Further, patients with COVID-19 also experience bleeding issues due to thrombocytopenia.

## Background:

The severe acute respiratory syndrome coronavirus-2 (SARS-CoV-2) outbreak continues to place a significant strain on healthcare systems, economies, and patient management. More than sixteen million people worldwide had contracted the highly contagious COVID-19 illness by the end of July 2020 [1]. In India, the influenza virus spread quickly in July 2020, with an estimated 496,988 active cases and 33,425 fatalities. Delhi, India's capital, and Mumbai, its financial center, accounted for 40% of all deaths caused by this widespread virus [2]. The 2019 new coronavirus disease (COVID-19) has been linked to increased morbidity and mortality, particularly in elderly individuals and those with diabetes, hypertension, obesity, and cardiovascular disorders [3]. It may cause severe acute respiratory syndrome. Furthermore, COVID-19 has a number of non-respiratory side effects, such as systemic side effects like thrombosis, septic shock, and disseminated intravascular coagulopathy (DIC) [4]. There have been numerous cases of COVID-19 pneumonia that have been associated with hematologic abnormalities, including lymphopenia, thrombocytopenia, and haemostatic anomalies. They are connected to ICU hospitalization, the severity of the illness and mortality [5]. When blood coagulation takes place, fibrin is released in the soluble state, releasing the D-Dimer that is contained in them into the blood. Therefore, an increase in D-Dimer levels is associated with the activation of blood coagulation, which facilitates diagnostic and therapeutic measures [6]. Infections like the flu are linked to higher D-Dimer levels, according to earlier research, because respiratory viruses activate the coagulation process [7]. The first might anticipate an increase in D-Dimer levels in COVID-19 owing to systemic inflammation and cytokine storm, but it can become quite important as the disease progresses due to internal coagulation [8]. Disseminated intravascular coagulation (DIC), in which D-Dimer levels were shown to be elevated, is virtually usually related with COVID-19 infection and acute respiratory distress syndrome (ARDS) [9]. Evaluation of D-Dimer levels is used to forecast the onset of ARDS in COVID-19 patients, which is most likely caused by a micro pulmonary embolism. Therefore, it is of interest to evaluate the role of D-Dimer and haematological parameters in patients with COVID 19.

## Materials and Methods:

This follow up study was conducted in department of pathology collaborated with medicine in Haveri Institute of Medical Sciences and Research center". The patients were diagnosed with SARS-COVID 19 by quantitative estimation. Inclusion criteria of the study subjects are age between 30 to 70 years and should be diagnosed with COVID 19. Whoever not is willing to participate, whoever not comes for 6 months follow up were excluded from the study. All the study subjects were recruited after Institutional Ethics Committee (IEC) permission and patient consent form.

## Sample collection:

Naso pharyngeal and oro-pharyngeal samples was collected with the help of swabs and transferred into viral transport media, precede immediately for quantitative analysis of COVID 19. Later on, we collected 5 mL of blood samples from all the study subjects, transferred 3ml into Ethylene Diamino Tetra Acetic Acid (EDTA) and remaining 2 ml transferred into sodium citrate vacutainer for Hb, RBCs, WBCs, PT, APTT, D-Dimer.

## Statistical Analysis:

The complete statistical analysis was done by using Microsoft excel spread sheets and statistical package for social science. The distribution of data checked by using kolmogorov Smirnov test and comparison of the variables done by using analysis of variance (ANOVA). The Pearson correlation analysis was used to correlate between the variables. The box plots were constructed variables between the groups.

## Results:

A total of 100 patients with COVID 19 were recruited and their clinical characteristics shown in Table 1. The age, Hb, RBCs, WBCs, PT, APTT, D-Dimer and CT of the patients were normally distributed, respectively the P value is >0.05. The mean levels of D-Dimer shown significantly increased in newly diagnosed COVID 19 when compared to after 6 months of follow up, respectively the P- value is 0.001**. The WBCs and haemoglobin mean values are shown significant values between the study subjects, respectively P- values are 0.001**. Along with that the PT and APTT significantly increased in newly diagnosed COVID 19 patients when compared to after 6 months of follow up, the P - value is 0.001**. The cycle threshold (CT) was very low in newly diagnosed COVID 19 when compared to after 6 months, the respectively P values less than 0.001**. The age and RBCs does not showed any significant between the study subjects, respectively P values are 0.98 and 0.81.

Table 2 illustrates the Pearson correlation analysis between the variables among study subjects. There was a positive correlation of WBCs, PT, APTT (r= 0.458, 526, 509) with D-Dimer and negatively correlated RBCS, Hb, CT (-0.056, 321, 526, 353), respectively P= 0.001**.

Figure 1(see PDF) shows, APTT and PT concentrations in study subjects. There was significantly increased levels of APTT and PT both patients with newly diagnosed COVID 19 and after follow up 6 months patients was identified.

Figure 2(see PDF) shows, WBCs, RBCs and Hb concentrations in study subjects. There was a significantly decreased level of WBCs, RBCs and Hb both newly diagnosed COVID 19 patients and after follow up 6 months patients was identified.

Figure 3 (see PDF) shows, D-Dimer and cycle threshold concentrations in study subjects. There was a significantly increased level of D-Dimer in patients with newly diagnosed COVID 19 when compared to after 6 months of follow up patients was identified. Additionally, decreased levels of cycle threshold in after 6 months of follow up patients than newly diagnosed COVID 19 patients.

## Discussion:

This study showed how the severity and course of COVID-19 patients' condition were affected by discrepancies in CBC parameters. CBC values were found to be abnormal, including increased WBC (leucocytosis), a sign of COVID-19 infection. In COVID-19 patients, a high WBC count has been found. Furthermore, compared to mildly infected COVID-19 patients, some studies also noted elevated WBC counts in severely infected ICU patients [10]. In contrast to the matched healthy controls, the ICU-admitted COVID-19 patients had a low haemoglobin concentration and RBC count. In fact, it has been proposed that neutrophilia and lymphopenia are related to the severity and mortality in COVID-19 patients [11]. Multiple research investigations have noted the higher risk of anaemia in people with severe COVID-19 disease and the link between anaemia and poor patient outcomes. We also observed that there was a significantly decreased level of haemoglobin in newly diagnosed COVID 19 patients when compared this concentrations with after 6 months of follow up. Additionally, in line with earlier research' findings, the current investigation revealed higher D-dimer levels in COVID-19 patients with extended PT and APTT. Previous studies are reported around 30% of the patients, who were primarily older patients with concomitant conditions and non survivors, had thrombocytopenia [12-13]. When compared to patients less than 60 years of age, patients over 60 years of age had longer PTs and higher INRs, and PTs longer than 27 seconds were also deadly in 42% of cases [14]. Thrombocytopenia and abnormal coagulation markers (such as PT, INR, and D-dimer) may be crucial signs of severe COVID-19 that are linked to death. In COVID-19 patients, elevated D-dimer levels have been associated with severity and mortality. D-dimers are generated through fibrinolysis, which entails tearing down fibrin clots; hence higher concentrations in COVID-19 patients indicate a propensity for thrombosis [15]. It has been hypothesized that elevated D-dimer levels are associated with conditions adverse effects, including the severity of COVID-19. In hospitals, elevated D-dimer levels have been shown to be an imitation and dependable predictive predictor of higher mortality among COVID-19 patients, facilitating early intervention strategies for COVID-19 patients [16]. Increases in D-Dimer, a so-called indirect marker of thrombus formation, in COVID-19 patients, especially those with ARDS patients, predict the development of thrombus and a bad prognosis [17]. However, a significant rise in D-Dimer suggested benefit from heparin infusion in a sizable cohort of ARDS patients as an indirect marker. There are a number of possible explanations for the substantial correlations that have been identified between CBC values and potential confounders. The severity of COVID-19 is likely indicated by the relationship between age and changes in the parameters of platelets and WBCs (mostly lymphopenia) [18]. The WBCs and RBCs are negatively correlated with cycle of threshold; Patients with severe COVID-19 also exhibited low PLT, high PT, and INR levels, which were linked to a poor prognosis. Mortality and comorbidities were strongly correlated with the aberrant pattern of coagulation markers.

## Conclusion:

The severity of COVID-19 cases and coagulation malfunction are strongly associated. They both have an impact on the prognosis of COVID-19 patients. Low platelet, high d-dimer, and fibrinogen may serve as risk markers for the progression of COVID-19 severity in the early screening of both severe and non-severe COVID-19 patients. COVID-19 patients may experience anomia-related consequences as hypoxia and coronary and pulmonary failure due to low Hb concentration. Further, patients with COVID-19 may also experience bleeding issues due to thrombocytopenia.

## Figures and Tables

**Table 1:** Comparison of clinical characteristics among study participants

**Parameters**	**Groups**	**Mean**	**Std. Deviation**	**P - Values**
Age	Newly Diagnosed COVID 19	51.47	13.47	0.98
	After Month	51.52	14.32	
D-Dimer	Newly Diagnosed COVID 19	1707.36	483.34	0.001**
	After Month	766.34	140.83	
WBCs	Newly Diagnosed COVID 19	5.4	0.72	0.001**
	After Month	4.66	0.42	
RBCs	Newly Diagnosed COVID 19	4.85	0.62	0.81
	After Month	4.87	0.42	
Hb	Newly Diagnosed COVID 19	7.31	1.66	0.001**
	After Month	8.44	0.91	
PT	Newly Diagnosed COVID 19	17.79	1.45	0.001**
	After Month	13.92	2.96	
APTT	Newly Diagnosed COVID 19	47.53	6.83	0.001**
	After Month	34.18	9.52	
CT	Newly Diagnosed COVID 19	20.03	4.23	0.001**
	After Month	24.76	4.59	

**Table 2:** Pearson's correlation analysis between the variables among study subjects

		**D-Dimer**	WBC_s_	**RBC_s_**	**Hb**	**PT**	**APTT**	**CT**
D-Dimer	Pearson Correlation	1	.458**	-0.056	-.321**	.526**	.509**	-.353**
	Sig. (2-tailed)		0	0.433	0	0	0	0
WBCs	Pearson Correlation	.458**	1	0.004	-0.094	.425**	.356**	-0.137
	Sig. (2-tailed)	0		0.955	0.186	0	0	0.053
RBCs	Pearson Correlation	-0.056	0.004	1	-0.032	-0.063	0.001	-0.076
	Sig. (2-tailed)	0.433	0.955		0.658	0.377	0.985	0.284
Hb	Pearson Correlation	-.321**	-0.094	-0.032	1	-.258**	-.224**	.200**
	Sig. (2-tailed)	0	0.186	0.658		0	0.001	0.005
PT	Pearson Correlation	.526**	.425**	-0.063	-.258**	1	.362**	-.254**
	Sig. (2-tailed)	0	0	0.377	0		0	0
APTT	Pearson Correlation	.509**	.356**	0.001	-.224**	.362**	1	-.277**
	Sig. (2-tailed)	0	0	0.985	0.001	0		0

## References

[1] Al-Jubury KS (2023). Braz J Biol..

[2] Mohammed Omer SA (2022). Pak J BiolSci.

[3] Ruetzler K (2021). Kardiol Pol.

[4] Du WN (2021). Int J Clin Pract.

[5] Hafeez MU (2021). Clin Appl Thromb Hemost.

[6] Kwaan HC (2022). Thromb Haemost.

[7] Ali AM (2022). Immun Inflamm Dis.

[8] Goudouris ES (2021). J Pediatr.

[9] Li C (2020). Acad Emerg Med.

[10] Elberts SJ (2021). Acad Emerg Med.

[11] Kalużna-Oleksy M (2021). Kardiol Pol.

[12] Korkusuz R (2021). Bratisl Lek Listy.

[13] Ettorre G (2022). J Med Virol.

[14] Mishra Y (2020). Diabetes Metab Syndr.

[15] Chen Z (2021). Clin Chim Acta.

[16] Conte G (2021). Clin Appl Thromb Hemost.

[17] Mei H (2020). J Hematol Oncol.

[18] Townsend L (2021). J Thromb Haemost.

